# Performance of Transient Elastography for the Staging of Liver Fibrosis in Patients with Chronic Hepatitis B: A Meta-Analysis

**DOI:** 10.1371/journal.pone.0044930

**Published:** 2012-09-25

**Authors:** Young Eun Chon, Eun Hee Choi, Ki Jun Song, Jun Yong Park, Do Young Kim, Kwang-Hyub Han, Chae Yoon Chon, Sang Hoon Ahn, Seung Up Kim

**Affiliations:** 1 Department of Internal Medicine, Yonsei University College of Medicine, Seoul, Korea; 2 Institute of Gastroenterology, Yonsei University College of Medicine, Seoul, Korea; 3 Liver Cirrhosis Clinical Research Center, Seoul, Korea; 4 Department of Biostatics, Yonsei University College of Medicine, Seoul, Korea; The University of Hong Kong, Hong Kong

## Abstract

**Background:**

Transient elastography (TE), a **non-invasive tool** that measures liver stiffness, has been evaluated in meta-analyses for effectiveness in assessing liver fibrosis in European populations with chronic hepatitis C (CHC). However, these data cannot be extrapolated to populations in Asian countries, where chronic hepatitis B (CHB) is more prevalent. In this study, we performed a meta-analysis to assess the overall performance of TE for assessing liver fibrosis in patients with CHB**.**

**Methods:**

Studies from the literature and international conference abstracts which enrolled only patients with CHB or performed a subgroup analysis of such patients were enrolled. Combined effects were calculated using area under the receiver operating characteristic curves (AUROC) and diagnostic accuracy values of each study.

**Result:**

A total of 18 studies comprising 2,772 patients were analyzed. The mean AUROCs for the diagnosis of significant fibrosis (F2), severe fibrosis (F3), and cirrhosis (F4) were 0.859 (95% confidence interval [CI], 0.857–0.860), 0.887 (95% CI, 0.886–0.887), and 0.929 (95% CI, 0.928–0.929), respectively. The estimated cutoff for F2 was 7.9 (range, 6.1–11.8) kPa, with a sensitivity of 74.3% and specificity of 78.3%. For F3, the cutoff value was determined to be 8.8 (range, 8.1–9.7) kPa, with a sensitivity of 74.0% and specificity of 63.8%. The cutoff value for F4 was 11.7 (range, 7.3–17.5) kPa, with a sensitivity of 84.6% and specificity of 81.5%.

**Conclusion:**

TE can be performed with good diagnostic accuracy for quantifying liver fibrosis in patients with CHB.

## Introduction

Liver fibrosis occurs in response to almost all causes of chronic liver insult, and its initiation is an important phase of chronic liver disease. [1] Without appropriate intervention, liver fibrosis progresses, leading to changes in liver morphology and deterioration of liver function and hemodynamics. Eventually, progression of liver fibrosis increases the risk for hepatocellular carcinoma (HCC) and hepatic decompensation, which are serious complications in patients with end-stage liver disease. [2], [3] Therefore, estimating the precise degree of liver fibrosis is important for predicting prognosis, surveillance, and optimizing treatment strategies in patients with chronic liver disease.

Liver biopsy (LB) is currently the gold standard for assessing liver fibrosis, but this invasive procedure may cause discomfort and pain, and rarely causes serious complications such as bleeding or biopsy-related mortality. [4] In addition, sampling errors and inter- and intraobserver variability may impede diagnostic accuracy. [5], [6] Despite these pitfalls, LB remains the standard diagnostic tool due to the absence of better alternatives. Therefore, many investigators have focused on the development and evaluation of noninvasive liver fibrosis assessment methods to replace LB. [7]–[9] Liver stiffness measurement (LSM) using transient elastography (TE; FibroScan®; Echosens, Paris, France) has been introduced as a promising noninvasive device for assessing liver fibrosis, with considerable accuracy and reproducibility for predicting cirrhosis. [10], [11].

Because TE was first developed in France, most studies of its benefits have been performed in European countries where chronic hepatitis C (CHC) is prevalent. Accordingly, extensive data on the clinical roles of TE in assessing liver fibrosis in patients with CHC have been gathered. [12]–[14] Furthermore, several meta-analyses have recently reported that TE is a reliable noninvasive tool to detect advanced liver fibrosis and cirrhosis. [15]–[18] However, because most studies included in the meta-analyses investigated European populations with CHC, these data cannot be extrapolated to populations in Asian countries where hepatitis B virus (HBV) infection is more prevalent than hepatitis C virus (HCV).

In recent years, the performance of TE has been evaluated in Asian patients with chronic hepatitis B (CHB),[19], [20] and the experiences of TE in these populations have accumulated. However, the overall performance of TE in patients with CHB has not been reported. Hence, in this study, we performed a meta-analysis to assess the overall performance of TE for the diagnosis of liver fibrosis in patients with CHB.

## Materials and Methods

### Transient Elastography

TE is a novel device for obtaining images using ultrasound. [21] TE is performed on the right lobe of the liver through the intercostal spaces on patients lying in the dorsal decubitus position with the right arm in maximal abduction. The operator locates a liver portion that is at least 6 cm thick and free of large vascular structures, and presses the transducer probe button to start the measurement. A vibration from the probe toward the tissue induces an elastic shear wave that propagates through the tissue. The velocity of the pulse-echo ultrasound following this propagation is measured; velocity increases with liver stiffness. The success rate is calculated by dividing the number of valid measurements by the total number of measurements. The interquartile range (IQR) is an index of intrinsic variability of TE, expressed as the interval of LSM results containing 50% of valid measurements between the 25th and 75th percentiles. The median value of successful measurements is selected as a representative LSM result, expressed in kilopascals (kPa).

### Literature Search

We searched medical databases (PubMed [MEDLINE], EMBASE, the Cochrane Library, and Google Scholar) to identify articles published between 2002 (when TE was first introduced) and March 2011. The search terms used were “FibroScan,” “transient elastography,” “elastography and liver,” “liver stiffness,” and “liver fibrosis.” In addition, we scanned the websites and conference abstract books of the American Association for the Study of the Liver, European Association for the Study of the Liver, Digestive Disease Week, and Asian Pacific Association for the Study of the Liver.

### Inclusion and Exclusion Criteria

For inclusion in this meta-analysis, studies needed to satisfy the following criteria: 1) enrolled only patients with CHB or performed a sub-group analysis of such patients; 2) evaluated the performance of TE to establish liver fibrosis stages based on LB as a reference standard; 3) used a comparable LB staging system, such as METAVIR or the systems of Ishak, Brunt, Ludwig, Knodell, Desmet, or Scheuer; and 4) evaluated the diagnostic accuracy of TE in assessing liver fibrosis stages using area under the receiver operating characteristic curves(AUROCs) and/or expressed sensitivity, specificity, positive predictive values (PPVs), or negative predictive values (NPVs) for the diagnosis of fibrosis stage based on certain cutoff TE values.

Studies were excluded from this analysis if they did not include patients with CHB, did not use LB as a reference test, did not use a fibrosis staging system comparable with METAVIR, and/or did not report AUROC values or diagnostic indices such as sensitivity, specificity, PPVs, or NPVs. Abstracts with data that were subsequently published as full-length articles or that obviously presented data from the same study at different meetings (same study group and patient population, identical study design, and same or increased number of patients) were evaluated, and only the most recent abstracts were included in this analysis. Reports not written in English were also excluded. Data extraction was performed independently by two reviewers (Chon YE and Kim SU), and when discrepancies surfaced, a final consensus opinion was adopted after discussion.

### Data Analysis

Data and results of the included studies are summarized in **Tables 1** and **2**. For meta-analysis, AUROC values were obtained from all included studies and the standard error of each study was determined or approximated from the available data using a 95% confidence interval (CI). To calculate the mean AUROC, scores from various fibrosis staging systems were standardized using a scale ranging from 0 to 4 points. For example, Ishak scores (0–6) were transformed to METAVIR scores, with Ishak ≥ F3 assigned to METAVIR ≥ F2, Ishak ≥ F4 assigned to METAVIR ≥ F3, and Ishak ≥ F5 assigned to METAVIR F4, respectively.

**Table 1:** Characteristics of studies evaluating the performance of transient elastography for staging liver fibrosis.

Author	Year	Country	Patients(n)	Final samplesize (n)	Failure		Male (%)	Mean BMI(kg/m^2^)	Etiology of disease
					LSM (reason)	LB (reason)			
Chan^24^	2009	China	186	161	1(SR<60%, <10VM)	22 (<15mm, <6pt)	76.0	24.0	HBV
Marcellin^25^	2009	France	202	173	14 (SR<50%, <7VM)	15 (<10pt)	66.5	24.5	HBV
Wong^26^	2008	China	182	133	10 (SR<60%, <10VM, IQR/M<0.3)	37 (<15mm, <6pt)	70.0	25.0	HBV, HCV, NAFLD, AIH, PBC
Kim^27^	2010	Korea	235	200	5 (SR<60%, <10VM)	10 (<15mm)	71.5	23.4	HBV
Wang^28^	2009	China	364	320	8 (SR<65%, <10VM)	36 (<10mm)	62.2	24.4	HBV, HCV
Kim^19^	2009	Korea	194	91	0 (SR<60%, <8VM)	4 (<10mm, <10pt)	80.2	23.8	HBV
Kim^29^	2009	Korea	130	130	0 (SR<60%, <10VM)	0 (<10mm, <6pt)	79.2	25.3	HBV
Sporea^30^	2010	Italy	140	140	0 (SR<60%, <10VM, IQR/M<0.3)	0 (N/A)	77.9	N/A	HBV, HCV
Jeon^31^	2007	Korea	45	45	0 (N/A)	0 (N/A)	N/A	N/A	HBV, HCV
Chang^32^	2007	Singapore	35	33	2 (obesity, narrow ICS)	0	N/A	25.6	HBV
Tawandee^33^	2008	Thailand	104	104	0 (N/A)	0 (N/A)	63.0	23.6	HBV
Choi^34^	2008	Korea	48	48	0 (N/A)	0 (N/A)	58.3	23.3	HBV
Castera^35^	2009	France	60	60	0 (N/A)	0 (N/A)	N/A	N/A	HBV
Chang^36^	2009	Singapore	88	84	3 (N/A)	1 (N/A)	71.6	N/A	HBV
Jia^37^	2010	China	486	486	0 (N/A)	0 (N/A)	N/A	22.0	HBV
Lesmana^38^	2010	Indonesia	62	62	0 (N/A)	0 (N/A)	N/A	22.8	HBV
Chen^39^	2011	China	389	315	0 (N/A)	0 (N/A)	N/A	N/A	HBV
Zhu^40^	2011	China	178	175	0 (N/A)	0 (N/A)	N/A	N/A	HBV

LSM, liver stiffness measurement; LB, liver biopsy; BMI, body mass index; SR, success rate; VM, valid fibroscan measurement; IQR, interquartile range; M, median; ICS, intercostal space; HBV, hepatitis B virus; HCV, hepatitis C virus; NAFLD, non-alcoholic fatty liver disease; AIH, autoimmune hepatitis; PBC, primary biliary cirrhosis.

**Table 2:** Diagnostic indices of studies evaluating the performance of transient elastography for staging liver fibrosis.

	METAVIR and other scoring system F ≥2				METAVIR and other scoring system F ≥3				METAVIR and other scoring system F = 4			
Study	AUROC	95% CI	Cut-off (kPa)	Sensitivity/ Specificity (%)	AUROC	95% CI	Cut-off(kPa)	Sensitivity/ Specificity (%)	AUROC	95% CI	Cut-off(kPa)	Sensitivity /Specificity (%)
Chan^24^	N/A	N/A	N/A	**N/A/N/A**	0.87	0.78–0.92	8.4	**84/76**	0.93	0.89–0.97	9.0	**98/75**
Marcellin^25^	0.81	0.73–0.86	7.2	**70/83**	0.93	0.88–0.96	8.1	**86/85**	0.93	0.82–0.98	11.0	**93/87**
Wong^26^	N/A	N/A	N/A	**N/A/N/A**	N/A	N/A	N/A	**N/A/N/A**	0.86	0.78–0.94	13.4	**91/79**
Kim^27^	N/A	N/A	N/A	**N/A/N/A**	N/A	N/A	N/A	**N/A/N/A**	0.85	0.80–0.90	N/A	**N/A/N/A**
Wang^28^	0.86	0.77–0.93	8.0	**80/77**	0.88	0.79–0.94	N/A	**N/A/N/A**	0.89	0.81–0.95	10.0	**85/88**
Kim^19^	N/A	N/A	N/A	**N/A/N/A**	N/A	N/A	N/A	**N/A/N/A**	0.80	0.69–0.92	9.7	**82/62**
Kim^29^	N/A	N/A	N/A	**N/A/N/A**	N/A	N/A	N/A	**N/A/N/A**	0.84	0.77–0.91	10.1	**76/81**
Sporea^30^	N/A	N/A	7.0	**59/70**	0.75	N/A	8.8	**53/85**	0.97	N/A	13.6	**86/99**
Jeon^31^	N/A	N/A	N/A	**N/A/N/A**	0.79	N/A	N/A	**N/A/N/A**	0.86	N/A	11.5	**86/78**
Chang^32^	0.66	N/A	11.8	**90/78**	N/A	N/A	N/A	**N/A/N/A**	N/A	N/A	14.5	**86/92**
Tawandee^33^	0.76	0.66–0.84	6.9	**70/79**	0.79	0.70–0.87	N/A	**N/A/N/A**	N/A	N/A	7.3	**93/61**
Choi^34^	0.88	0.76–1.00	7.7	**88/88**	0.86	0.75–0.97	N/A	**N/A/N/A**	0.86	0.75–0.97	10.4	**79/83**
Castera^35^	0.76	0.63–0.89	N/A	**N/A/N/A**	N/A	N/A	N/A	**N/A/N/A**	0.89	0.80–0.98	N/A	**N/A/N/A**
Chang^36^	0.80	0.71–0.89	8.8	**N/A/N/A**	N/A	N/A	N/A	**N/A/N/A**	N/A	N/A	N/A	**N/A/N/A**
Jia^37^	0.82	0.78–0.85	7.3	**66/83**	0.88	0.84–0.91	9.7	**73/90**	0.90	0.87–0.94	17.5	**60/93**
Lesmana^38^	0.70	0.57–0.83	6.1	**71/65**	N/A	N/A	N/A	**N/A/N/A**	N/A	N/A	N/A	**N/A/N/A**
Chen^39^	0.87	0.83–0.91	N/A	**N/A/N/A**	0.89	0.86–0.93	N/A	**N/A/N/A**	0.89	0.85–0.93	N/A	**N/A/N/A**
Zhu^40^	0.95	0.91–0.98	7.9	**N/A/N/A**	N/A	N/A	N/A	**N/A/N/A**	0.98	0.96–0.99	13.8	**N/A/N/A**

AUROC, area under receiver operating characteristic curve; CI, confidence interval; kPa, kilopascal; **N/A, not available.**

Using AUROC and sensitivity values from each study, we performed a homogeneity test for each effect. Heterogeneity resulting from the effects of many different factors (different staging systems, different LB skills and lengths of specimen, TE skills and failure rates, and different patient demographics) existed among the studies. Therefore, we evaluated the significance of the estimated combined effects using a random effects model [22], which addressed the heterogeneity of studies in analyzing the efficacy of TE. The quality of the studies included in the meta-analysis was assessed by the Quality Assessment of Studies of Diagnostic Accuracy Included in Systematic Review (QUADAS) questionnaire (**Table S1**)and each item was rated as yes, no, or unclear. [23].

## Results

### Selection of Candidate Studies

The literature search identified 52 primary studies (30 full-length articles and 22 abstracts) that evaluated the performance of TE. However, several studies were excluded because they did not include patients with CHB (13 studies), did not provide data for diagnostic accuracy (AUROC; 6 studies), did not provide sensitivity or specificity values for any fibrosis stage (6 studies), were not written in English (3 studies), did not used LB as a reference (3 studies), and/or used a fibrosis staging system that was not comparable with METAVIR (3 studies). Finally, 18 studies[19], [24]–[40] were included in the meta-analysis after reading each study in full and adopting inclusion and exclusion criteria. All eighteen studies fulfilled >10/14 QUADAS items and successfully passed the quality assessment.

### Patient Characteristics and Study Results

The patient characteristics and results of studies chosen for meta-analysis are summarized in **Table 1**. The median sample size was 159 (range, 35–486) patients, and 18 studies comprising 2,772 patients were included in the analysis. The mean age was 44.8 (range, 35.6–57.8) years, and 48.6% of the patients were men. The fibrosis staging systems used to classify liver histology varied. Fourteen studies (77.8%) used the METAVIR score, 2 (11.0%) studies used the Batts and Ludwig scores, 1 (5.6%) study used the Scheuer score, and 1 (5.6%) study used the Ishak score. Fourteen (77.8%) of the 18 studies enrolled only patients with CHB, and 10 (55.6%) studies demonstrated all indices of diagnostic accuracy (sensitivity, specificity, PPV, and NPV) for all fibrosis stages.

### Meta-analysis of TE for Staging Liver Fibrosis

**Table 2** shows the AUROC, 95% CI, cutoff values, sensitivity, and specificity of each study. Based on these data, the meta-analysis was performed to obtain TE cutoff values for each liver fibrosis stage (**Table 3**).

**Table 3:** Meta-analysis results of LSM cutoff values for staging liver fibrosis.

	Patients (n)	Weighted MeanLSM value (kPa)	Range (kPa)	Sensitivity (%)	Specificity (%)
F ≥2	1,625	7.9	6.1–11.8	74.3	78.3
F ≥3	960	8.8	8.1–9.7	74.0	63.8
F = 4	2,051	11.7	7.3–17.5	84.6	81.5

LSM, liver stiffness measurement; kPa, kilopascal.

Ten (55.6%) studies provided cutoff values for predicting significant fibrosis (≥F2). Five studies reported all diagnostic accuracy parameters (sensitivity, specificity, PPV, and NPV), and the other five studies reported only sensitivity and specificity. A total of 1,625 patients were included in the meta-analysis and 7.9 kPa (range, 6.1–11.8 kPa; sensitivity, 74.3%; specificity, 78.3%) was determined as a cutoff value for predicting significant fibrosis. Based on the AUROC values and corresponding 95% CIs for predicting significant fibrosis of 10 (55.6%) studies, the combined AUROC value considering random effects was calculated as 0.859 (95% CI, 0.857–0.860) (**Figure 1a**).

**Figure:**
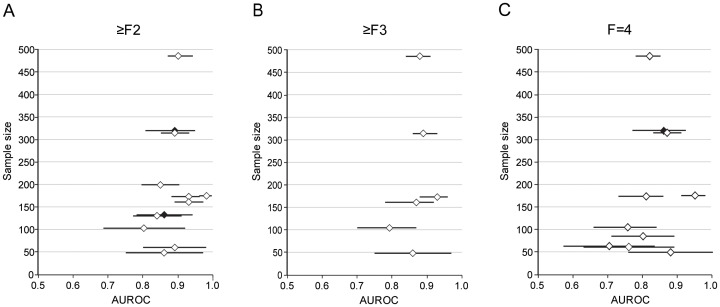
Forest plot from meta-analysis of AUROC value using a random-effect model for fibrosis stages (a) significant fibrosis (b) severe fibrosis (c) cirrhosis. The distribution is depicted according to the sample size and the length of the horizontal line represents the 95% CI. : AUROC of the studies with patients with CHB only. : AUROC of the studies with mixed etiologies with available sub-group analysis for patients with CHB.

Four (22.2%) studies provided cutoff values for predicting advanced fibrosis (≥F3). Two studies reported all diagnostic indices, whereas two reported only sensitivity and specificity. The cutoff value from 960 patients was determined as 8.8 kPa (range, 8.1–9.7 kPa; sensitivity, 74.0%; specificity, 63.8%). Based on the AUROC values and corresponding 95% CIs for predicting advanced fibrosis of six (33.3%) studies, the combined AUROC value considering random effects was 0.887 (95% CI, 0.886–0.887) (**Figure 1b**).

Thirteen (72.2%) studies were used to determine the cutoff values for cirrhosis (F4). Seven studies reported all diagnostic indices, whereas six reported only sensitivity and specificity. The cutoff value from 2,051 patients was determined as 11.7 kPa (range, 7.3–17.5 kPa; sensitivity, 84.6%; specificity, 81.5%). Based on the AUROC values and corresponding 95% CIs for predicting cirrhosis of 12 (66.7%) studies, the combined AUROC value considering random effects was 0.929 (95% CI, 0.928–0.929) (**Figure 1c**).

### Publication Bias

No outlier study and possible publication bias was identified in ≥F2, ≥F3, and F4, respectively (Figure 1a–c).

## Discussion

To overcome the limitations of LB in assessing the severity of liver fibrosis in patients with chronic liver disease, a great effort has been made to develop and validate noninvasive methods for detecting liver fibrosis. Among these noninvasive methods, TE has been widely studied as a novel noninvasive method of quantifying the degree of liver fibrosis, mainly in patients with CHC. [12], [41] Recently, the application of TE has also been extended to patients with CHB, based on cumulated evidence indicating accuracy comparable with that of CHC. [25], [42] Because a systematic approach is required for integrating the TE data from independent studies, we performed a meta-analysis to provide a combined systematic review of the accuracy of TE diagnostics in patients with CHB. In contrast to four previous meta-analyses that included individual studies comprising mostly patients with CHC,[15]–[18] our study tried to elevate the accuracy of the meta-analysis by exclusively selecting patients with CHB, thereby eliminating potential bias due to different viral etiologies.

The combined effect of AUROC values using a random-effect model[22] for the diagnosis of ≥F2, ≥F3, and F4 were 0.859 (95% CI, 0.857–0.860), 0.887 (95% CI, 0.886–0.887),and 0.929 (95% CI, 0.928–0.929) respectively, which are not excellent, but are acceptable and comparable to those from previous meta-analyses (**Table 4**). [43] Although the sensitivity and specificity of our study for predicting ≥F2 (74.3% and 78.3%, respectively) were similar to those from previous meta-analyses (70–79% and 78–84%, respectively), they were lower for predicting F4 (84.6% and 81.5%, respectively) than those from previous studies (83–87% and 89–91%, respectively; **Table 4**). This slightly lower sensitivity and specificity in our study can be explained in part by differences in the composition of the study population. In previous meta-analyses, the predominant etiology of chronic liver disease was chronic HCV infection. Indeed, the proportions of individual studies focusing on patients with CHC were 85.7% (18/21) in the study by *Stebbing et al.,* [16] 77.8% (7/9) in that by *Talwalkar et al.,* [15] and 42.5% (17/40) in the study by *Tsochatzis et al*.,[18] whereas our study included only patients with CHB. Compared with CHC, CHB displays a more complex natural history and frequent exacerbations accompanied by fluctuating alanine aminotransferase (ALT) levels. [44], [45] Therefore, overestimated TE values due to high ALT levels at the time of measurement might have produced false-positive results[27] and reduced the overall sensitivity and specificity of TE in our study.

**Table 4:** Characteristics of previous reported meta-analyses versus current study.

	Number of included studies	Number of included subjects for analysis	AUROC			Sensitivity/Specificity (%)		Cutoff values (kPa)		
			≥ F2	≥ F3	F4	≥ F2	F4	≥ F2	≥ F3	F4
Talwalkar^15^	**9**	**2,083**	0.870	N/A	0.957	70/84	87/91	N/A	N/A	N/A
Stebbing^16^	**22**	**4,760**	0.84	0.89	0.94	70/84	87/91	7.81	N/A	15.56
Fredrich-rust et al^17^	**50**	**8,206**	0.84	0.89	0.94	N/A	N/A	7.65	N/A	13.01
Tsochatzis et al^18^	**40**	**7,723**	N/A	N/A	N/A	79/78	83/89	7.3	10.2	15.0
**Chon et al**	**18**	**2,772**	**0.859**	**0.887**	**0.929**	**74.3/78.3**	**84.6/81.5**	**7.9**	**8.8**	**11.7**

AUROC, area under the receiver operating characteristic curve; kPa, kilopascal.

The optimal cutoff values in our study were 7.9 kPa for ≥F2, 8.8 kPa for ≥F3, and 11.7 kPa for F4. However, *Stebbing et al*. [16] determined higher cutoff values (7.81 kPa for ≥F2 and 15.56 kPa for F4), which increased further when calculated only for patients with CHC (8.44 kPa for ≥F2 and 16.14 kPa for F4). *Fredrich-rust et al*. [17] and *Tsochatzis et al.* [18] also adopted higher cutoff values (13.01 and 15.0 kPa, respectively) for determining F4 compared with that found in our study (11.7 kPa; **Table 4**). This tendency of low cutoff TE values in our study may be explained by two unique features of CHB. First, *Sturm et al.* [46] concluded recently that the total amount of liver fibrosis reflected by the fibrosis area was significantly lower in patients with CHB, because the fibrous septa might be thinner in these patients than in those with CHC with the same histological stage (F4). Second, because CHB tends to progress to cirrhosis with larger nodules (macronodular cirrhosis) than CHC, the TE pulse is more likely to pass through the normal liver parenchyma between fibrotic bands in patients with CHB than in those with CHC. [47] These two observations might have resulted in a lower cutoff TE value for patients with CHB compared with patients with CHC. Thus, physicians should be aware of the pitfalls of TE, such as false negativity or lower cutoff values resulting from macro nodular cirrhosis and thin fibrous septa, and false positivity or lower performance resulting from high ALT levels. [48] Accordingly, TE results should be interpreted within the clinical context.

Based on the results of our meta-analysis, TE seems to be a good tool for assessing liver fibrosis in patients with CHB, but it is not excellent. However, because LB is not a perfect gold standard, it is nearly impossible to achieve an AUROC close to 1 in an analysis based on LB data, even with a hypothetically perfect noninvasive liver fibrosis measurement tool. [49] Although TE is inferior to histological evaluation in principle, TE is superior to clinical diagnostic criteria in diagnosing compensated cirrhosis. [19], [50] Thus, the performance of TE in our study (AUROC = 0.93) for predicting cirrhosis is sufficiently accurate. Accurate evaluation of TE diagnostic performance will only be possible after establishing an optimal reference standard, such as laparoscopic biopsy from a designated liver location.

The presence of significant fibrosis (≥F2) has been regarded as an important indicator of chronic liver disease progression to grave prognosis. Therefore, intervention with antiviral treatment to stop or slow the disease progression is optimally performed at the time of identification of significant fibrosis in patients with chronic liver disease. [51], [52] However, the diagnostic accuracy of TE in predicting significant fibrosis seems imperfect in our study (AUROC = 0.86), as proved in previous reports examining patients with CHC. [15]–[17] Although the combined use of TE and other serological markers has been investigated to improve the diagnostic accuracy of distinguishing fibrosis stage ≥F2 from F0/1,[12], [53] this approach has been unsatisfactory, and histological evaluation still has a significant role in this condition.

Although the optimal reference cutoff values were established from our meta-analysis, a cross-sectional comparison between LB and TE values might be unsatisfactory in providing more clinical implications. Thus, recent studies have focused on the potential role of TE as an independent parameter in predicting the future clinical endpoint in longitudinal follow-up settings. Indeed, the TE value was significantly associated with the risk of HCC development[50], [54] or liver-related events. [55] In addition, TE has shown superiority to other serological fibrosis prediction models in predicting HCC or hepatic decompensation. [56] These data converge into a conclusion that TE can be used as a novel tool to help predict future prognosis in patients with chronic liver disease, although cannot fully replace LB as a diagnostic tool.

In conclusion, using the meta-analysis method, this systematic review focused on the performance of TE for detecting liver fibrosis in patients with CHB.TE seems to be a good method for quantifying hepatic fibrosis in these patients, and further longitudinal studies are required to validate our data.

## Supporting Information

Table S1 The QUADAS tool.
(DOC)
