# Supplementary material for: Performance of Transient Elastography for the Staging of Liver Fibrosis in Patients with Chronic Hepatitis B: A Meta-Analysis
Source: PLoS One. 2012 Sep 25;7(9):e44930. doi: 10.1371/journal.pone.0044930 (PMC3458028; doi:10.1371/journal.pone.0044930)
Supplement: Table S1 — The QUADAS tool. (DOC) [file pone.0044930.s001.doc]

| **Table S1.** The QUADAS tool |
| --- |
| 1. Was the spectrum of patients representative of the patients who will receive the test in practice? |
| 2. Was the spectrum of patients representative of the patients who will receive the test in practice? |
| 3. Is the reference standard likely to correctly classify the target condition? |
| 4. Is the time period between reference standard and index test short enough to be reasonably sure that the target condition did not change between the two tests? (disease progression bias) |
| 5. Did the whole sample, or a random selection of the sample, receive verification using a reference standard of diagnosis? (partial verification bias) |
| 6. Did patients receive the same reference standard regardless of the index test result? (differential verification bias) |
| 7. Was the reference standard independent of the index test (i.e. the index test did not form part of the reference standard? (incorporation bias) |
| 8. Was the execution of the index test described in sufficient detail to permit replication of the test? |
| 9. Was the execution of the reference standard described in sufficient detail to permit its replication? |
| 10. Were the index test results interpreted without knowledge of the results of the reference standard? (test review bias) |
| 11. Were the reference standard results interpreted without knowledge of the results of the index test? (diagnostic review bias) |
| 12. Were the same clinical data available when test results were interpreted as would be available when the test is used in practice? (clinical review bias) |
| 13. Were uninterpretable/intermediate test results reported? |
| 14. Were withdrawals from the study explained? |
| QUDAS, Quality Assessment of Studies of Diagnostic Accuracy Included in Systematic Review. |
